# Intermittent Hypoxia Can Aggravate Motor Neuronal Loss and Cognitive Dysfunction in ALS Mice

**DOI:** 10.1371/journal.pone.0081808

**Published:** 2013-11-26

**Authors:** Sung-Min Kim, Heejaung Kim, Jeong-Seon Lee, Kyung Seok Park, Gye Sun Jeon, Jeeheun Shon, Suk-Won Ahn, Seung Hyun Kim, Kyung Min Lee, Jung-Joon Sung, Kwang-Woo Lee

**Affiliations:** 1 Department of Neurology, Seoul National University, College of Medicine, Seoul, Korea; 2 Department of Neurology, Hanyang University, College of Medicine, Seoul, Korea; 3 Department of Pediatrics, Seoul National University, College of Medicine, Seoul, Korea; 4 Department of Neurology, Chung-Ang University Hospital, Chung-Ang University College of Medicine, Seoul, Republic of Korea; Institute of Health Science, China

## Abstract

**Background:**

Patients with ALS may be exposed to variable degrees of chronic intermittent hypoxia. However, all previous experimental studies on the effects of hypoxia in ALS have only used a sustained hypoxia model and it is possible that chronic intermittent hypoxia exerts effects via a different molecular mechanism from that of sustained hypoxia. No study has yet shown that hypoxia (either chronic intermittent or sustained) can affect the loss of motor neurons or cognitive function in an in vivo model of ALS.

**Objective:**

To evaluate the effects of chronic intermittent hypoxia on motor and cognitive function in ALS mice.

**Methods:**

Sixteen ALS mice and 16 wild-type mice were divided into 2 groups and subjected to either chronic intermittent hypoxia or normoxia for 2 weeks. The effects of chronic intermittent hypoxia on ALS mice were evaluated using the rotarod, Y-maze, and wire-hanging tests. In addition, numbers of motor neurons in the ventral horn of the spinal cord were counted and western blot analyses were performed for markers of oxidative stress and inflammatory pathway activation.

**Results:**

Compared to ALS mice kept in normoxic conditions, ALS mice that experienced chronic intermittent hypoxia had poorer motor learning on the rotarod test, poorer spatial memory on the Y-maze test, shorter wire hanging time, and fewer motor neurons in the ventral spinal cord. Compared to ALS-normoxic and wild-type mice, ALS mice that experienced chronic intermittent hypoxia had higher levels of oxidative stress and inflammation.

**Conclusions:**

Chronic intermittent hypoxia can aggravate motor neuronal death, neuromuscular weakness, and probably cognitive dysfunction in ALS mice. The generation of oxidative stress with activation of inflammatory pathways may be associated with this mechanism. Our study will provide insight into the association of hypoxia with disease progression, and in turn, the rationale for an early non-invasive ventilation treatment in patients with ALS.

## Introduction

Amyotrophic lateral sclerosis (ALS) is a disease of the central nervous system that manifests as progressive cognitive dysfunction and motor weakness due to the degeneration of the fronto-temporal lobes and motor neurons [1,2]. Although it is degeneration of the central nervous system that primarily causes ALS [3], several recent studies have suggested that hypoxia can also be involved in the aggravation of this disease [4-6]. 

Patients with ALS frequently experience hypoxia due to progressive weakness of the respiratory muscles [7] and/or dysfunction of central respiratory drive [8]. Although numerous in vitro studies have suggested adverse effects of hypoxia in ALS [9-12], no in vivo study has yet demonstrated the effects of hypoxia on the motor neuronal loss or symptom aggravation in ALS.

Patients with ALS can be exposed to 2 different types of hypoxia: chronic sustained hypoxia (CSH) and chronic intermittent hypoxia (CIH) [13]. These 2 types of hypoxia have been shown to selectively activate different molecular pathways [14]. To date, no studies have specifically investigated the effects of CIH in an experimental model of ALS. We hypothesized that CIH would aggravate motor weakness and/or cognitive function in ALS. 

## Methods

### ALS mice

B6SJL-Tg(SOD1-G93A) 1Gur/J mice, which have a glycine 93-(Gly93) to alanine (Ala) substitution in the superoxide dismutase 1 (SOD1) gene, were purchased from the Jackson Laboratory (Bar Harbor, ME, USA) and kept under a 12-h light/dark cycle and bred as per the supplier’s protocol [15]. DNA was extracted from the tail tissues and polymerase chain reaction (PCR) assays were performed to test for the presence of the human *G93A* transgene. Sixteen transgenic ALS mice (8 males, age 14 weeks) and 16 age- and sex-matched wild-type (Wt) control mice were divided equally into 2 groups: a CIH group and a normoxia (NOX) group. This study was approved by the IACUC (Institutional Animal Care and Use Committee) of Seoul National University.

### Intervention

**Figure 1:**
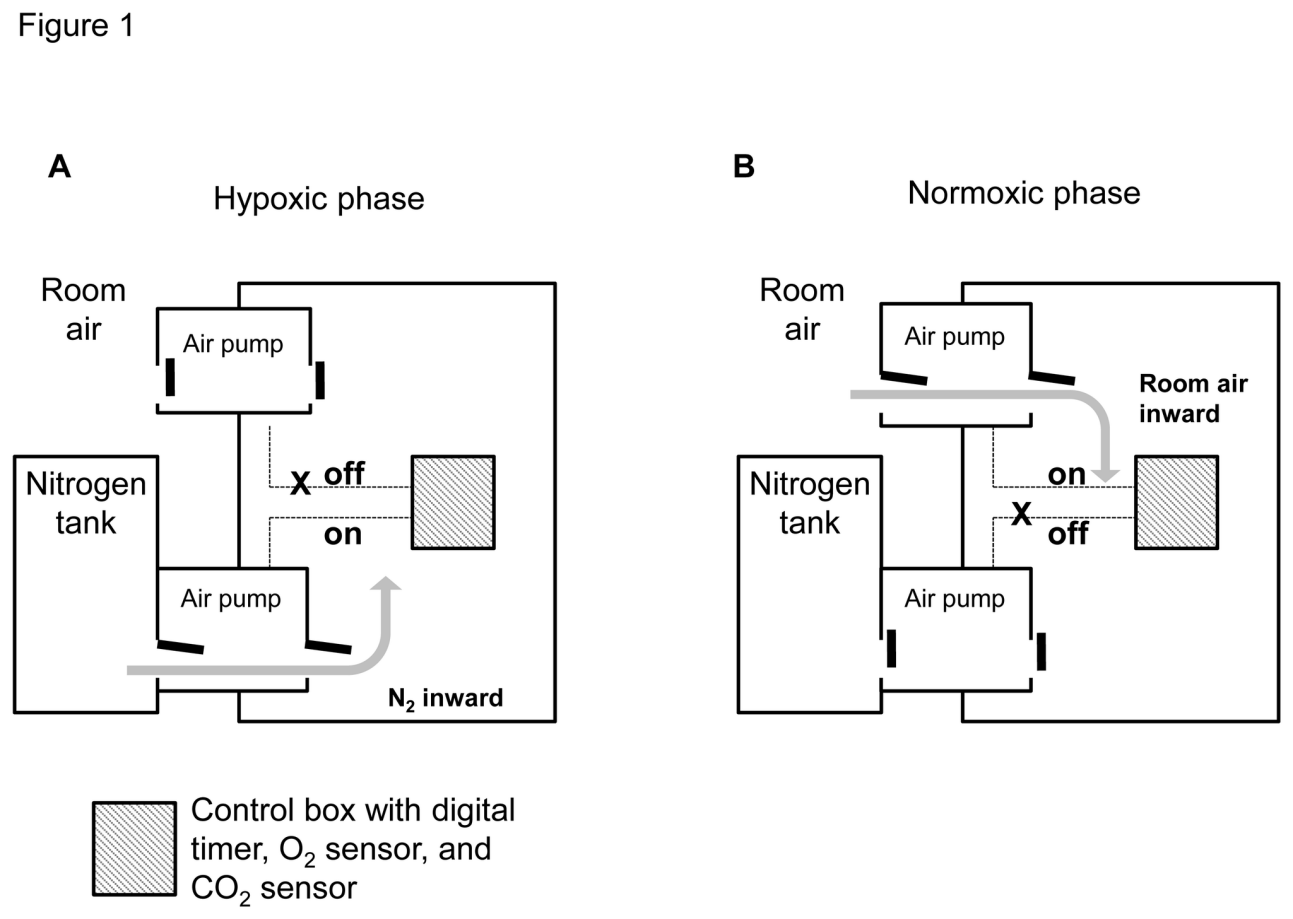
Diagrams illustrating the experimental setup for the hypoxic (A) and normoxic (B) phases. Customized semi-closed hypoxia chambers with solenoid valves were developed to achieve chronic intermittent hypoxia. The hypoxic phase (90 s) was achieved with rapid infusion of nitrogen gas and the normoxic phase (4 min) was achieved with rapid infusion of room air. The final F_i_O_2_ levels of the hypoxic and normoxic phases were 7.8% and 20%, respectively. In the CIH group, hypoxic and normoxic phases were repeated consecutively 12 h/day for a total of 2 weeks.

A semi-closed hypoxia chamber (VS-9108MS2; Vision Science Co., Seoul, Korea; 420 × 410 × 600 mm [width × diameter × height]) was modified to meet the specifications of this study. A customized air infusion pump with timer was connected to the chamber and 4 boxes (180 × 280 × 150 mm) were placed inside the chamber for rapid infusion of either nitrogen or room air. The hypoxic period (90 s, final F_i_O_2_ 7.8%) was achieved with rapid infusion of nitrogen gas and a normoxic period (4 min, final F_i_O_2_ 20%) was achieved with rapid infusion of room air (Figure 1). 

Mice in the CIH group were maintained with repetitive cycles of intermittent hypoxia and normoxia for 12 h/day for a total of 2 weeks. To exclude effects of temperature, moisture, and noise, mice in the NOX group were maintained in the same chamber (VS-9108MS2) for 12 h/day, but with only continuous room-air infusion, without the hypoxic periods.

### Behavioral tests

Rotarod testing was conducted for the evaluation of motor coordination and learning [16,17] After a training session of 2 consecutive days immediately before the first test session, mice were subjected to a total of 4 rotarod test sessions with accelerating speeds (range, 4–40 rpm) on Days 8, 9, 12, and 15 after initiation of CIH or NOX. Each test session was composed of 2 trials on the rotarod, each with a maximum duration of 5 min, and an inter-trial interval of 1 h. The retention time on the rotarod for each mouse was measured and the best result from each trial was used for further analysis. 

The wire-hanging test was performed to measure neuromuscular strength [17,18]. Mice were gently placed on a wire-cage lid, which was then slowly waved and turned upside down above the soft bedding. The hanging time for each mouse to fall onto the bedding below was measured with a cut-off time of 60 s.

The Y-maze spontaneous alternation test was performed to assess spatial memory, which is dependent on hippocampal function [19]. The Y-shaped maze consisted of 3 black plastic arms placed at 120° angles to each other. Mice were placed at the end of one arm and were allowed to explore the maze freely for 8 min without training, reward, or punishment. An alternation was defined as a complete cycle of consecutive entrances into each of the 3 arms. Percent alternation (PA) was calculated as follows: PA = number of alternations/(total number of entrances into each arm – 2) [19].

### Immunoblot analysis

Immunoblot analysis was used to quantify the expression of NF-κB inhibitor alpha (IκBα, Ab32518; Abcam, Cambridge, UK) [14], 4-hydroxynonenal (4-HNE, HNE 12-S; Alpha Diagnostic, San Antonio, TX, USA) [20], and gliofibrillary acidic protein (anti-GFAP; Santa Cruz Biotechnology, CA, USA). After behavioral testing, mice (four mice in ALS-CIH, 3 in ALS-NOX, 4 in Wt-CIH, and 4 in Wt-NOX) were anesthetized with pentobarbital sodium and perfused transcardially with cold phosphate-buffered saline (PBS). The spinal cord of each mouse was removed and frozen in a deep freezer (-80°C), as previously described [21]. Tissues were homogenized in tissue-protein extraction reagent (T-PER; Pierce/Thermo Fisher Scientific, Waltham, MA, USA) and centrifuged (13200 rpm, 20 min, 4°C). Supernatant was collected for the determination of protein concentration using the Bradford method, and was subsequently separated on SDS/polyacrylamide gels (Invitrogen SDS-PAGE; Life Technologies, Carlsbad, CA, USA). The intensities for IκBα and 4-HNE were quantified and analyzed as relative values to GFAP by using Image J.

### Motor neuron counts and immunohistochemistry

Mice were anesthetized with pentobarbital sodium and then perfused transcardially with cold PBS followed by cold paraformaldehyde. The spinal cords were dissected, fixed in paraformaldehyde, and cryoprotected in a cold sucrose solution. Spinal cord enlargement was used to identify the cervical portions of the spinal cords and 20-μm-thick transverse sections were obtained. Motor neurons in the ventral horn of the spinal cord were stained for choline acetyltransferase (anti-ChAT; Millipore, Billerica, MA, USA) and were counted, as previously described [22]. Activation of microglia and astrocytes were assessed using antibodies to ionized calcium-binding adapter molecule 1 (IBA1; Wako, Japan) and GFAP (Santa Cruz Biotechnology), respectively.

### Statistical analyses

Throughout the text, results are expressed as means ± standard deviations. Statistical analysis was performed using a Student’s *t*-test and one-way ANOVA followed by Fisher’s least significant difference post-hoc tests. The threshold for statistical significance was set at *p* < 0.05. 

## Results

### CIH impairs motor learning in ALS mice (Figure 2)

**Figure 2:**
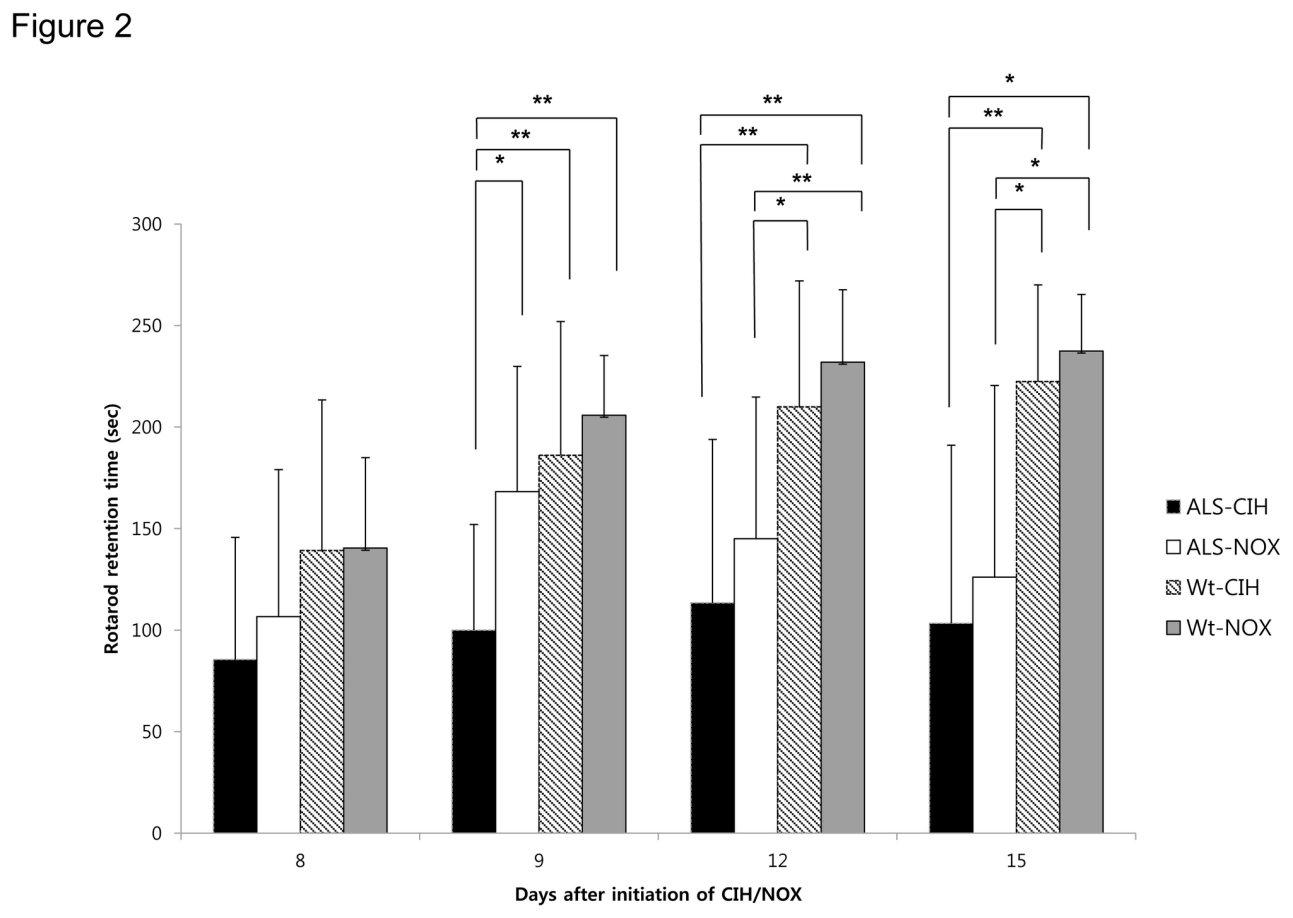
Performance on the rotarod test. The rotarod test was performed to evaluate motor coordination and motor learning on Days 8, 9, 12, and 15 after the initiation of CIH or NOX treatments. On the second day of rotarod testing (Day 9), the Wt and ALS-NOX mice showed marked improvement in rotarod retention times, compared to their performance on the first day of testing (Day 8). In contrast, compared with the ALS-NOX mice, the ALS-CIH mice did not show an improvement in rotarod retention times and had significantly shorter rotarod retention times on Day 9 (*p* < 0.05), which could be the results of impairment in motor learning ability. In addition, ALS-CIH mice tended to show shorter rotarod retention times than the ALS-NOX mice did throughout the serial follow-up period, although this trend did not reach statistical significance for Days 8, 12, or 15. * *p* < 0.05, **, *p* < 0.01.

In the Wt and ALS-NOX mice, motor learning was demonstrated by marked improvements in rotarod retention times on the second day of rotarod testing (Day 9), compared to the first day of rotarod testing (Day 8). However, in the ALS-CIH group, only minor improvements in rotarod retention times were observed on the second day of rotarod testing. The mean rotarod retention time on the second day of testing was significantly shorter in the ALS-CIH mice (100.00 ± 52.06 s) than in the ALS-NOX mice (168.25 ± 61.60 s, *p* = 0.017) and Wt-CIH mice (186.13 ± 65.84 s, *p* = 0.041). Compared to the Wt-NOX mice, the Wt-CIH mice showed a tendency toward shorter rotarod retention times throughout the consecutive test sessions; however, this trend did not reach statistical significance. After Days 12 or 15, ALS mice began to demonstrate reductions in rotarod retention times, which may have been due to their progressive muscle weakness. The rotarod retention times on Days 12 and 15 did not differ significantly between the ALS-CIH group and the ALS-NOX group.

### CIH impairs spatial memory in ALS mice (Figure 3)

**Figure 3:**
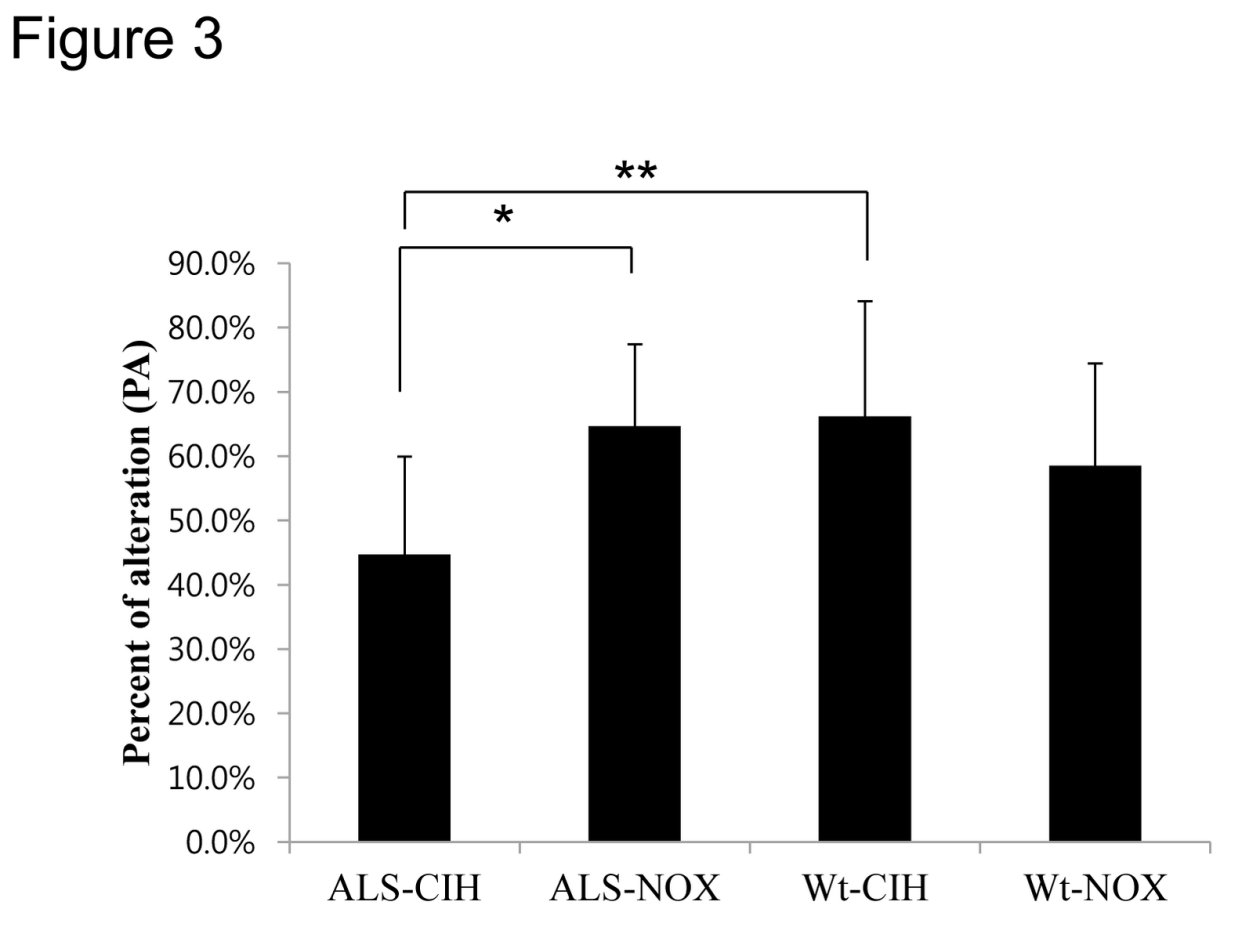
Percent alternation in the Y-maze test. The Y-maze test was performed to evaluate the effect of CIH on spatial memory in mice. After 2 weeks of CIH, ALS mice exhibited significantly lower percent alternation (PA) in the Y-maze test than did the ALS-NOX mice (*p* < 0.05). In addition, the ALS-CIH mice showed poorer spatial memory compared with the Wt-CIH mice, an effect which suggests that ALS mice are more vulnerable to CIH than the Wt mice are. * *p* < 0.05, **, *p* < 0.01.

PA was significantly lower in the ALS-CIH mice (44.8 ± 15.2%) than in the ALS-NOX mice (64.8 ± 12.7%, *p* = 0.016) and Wt-CIH mice (66.3 ± 17.9%, *p* = 0.010). However, PA did not significantly differ between the Wt-CIH and Wt-NOX mice (58.6 ± 15.9%, *p* > 0.05).

### CIH aggravated neuromuscular strength in ALS mice (Figure 4)

**Figure 4:**
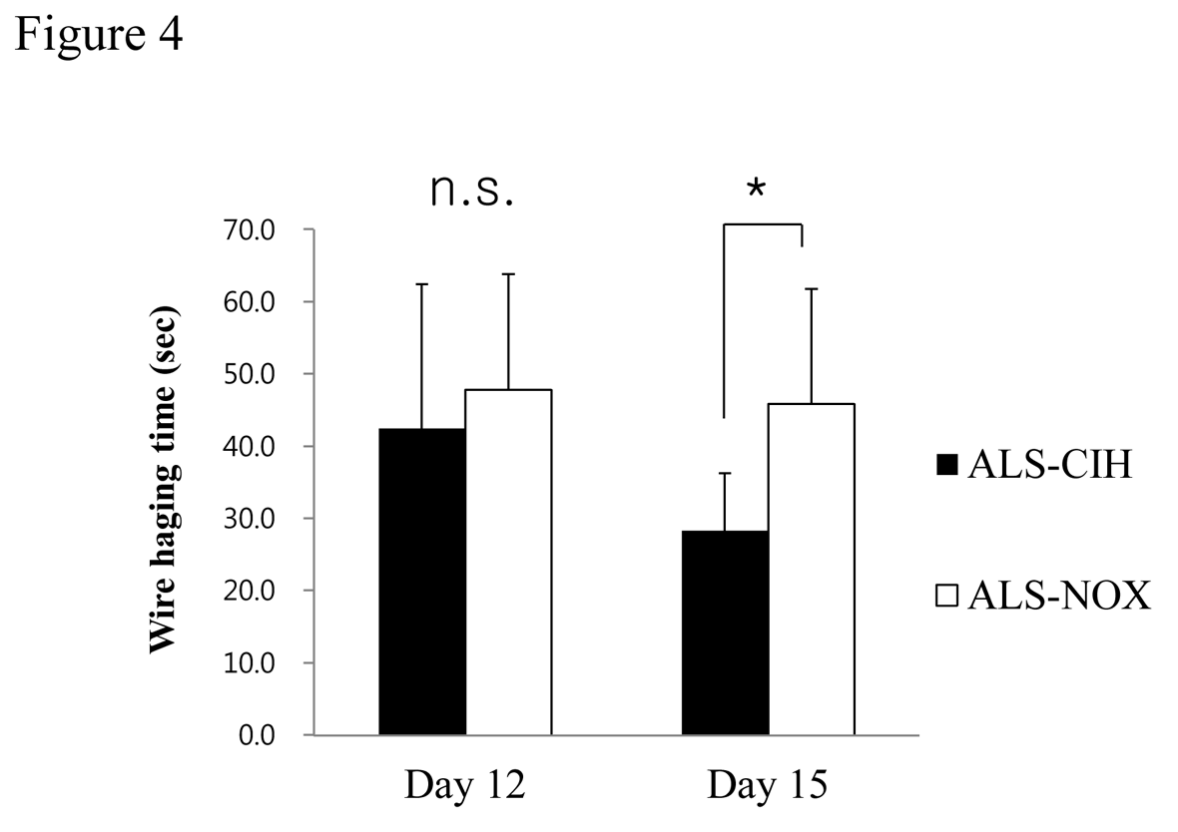
Wire-hanging test. Wire-hanging times were tested to measure the effect of CIH on neuromuscular strength in ALS mice. The ALS-CIH mice exhibited somewhat reduced neuromuscular strength compared to the ALS-NOX mice (*p* = 0.037). * *p* < 0.05.

Duration of wire hanging were measured and compared among mice that accomplished the wire hanging test (6 mice in each group). The ALS-CIH mice showed a significantly shorter wire hanging time compared to the ALS-NOX mice on Days 15 after initiation of CIH/NOX (28.3 ± 7.89 seconds vs. 45.8 ± 15.94, *p* = 0.037).

### CIH and facilitation of motor neuron death in ALS mice (Figure 5)

**Figure 5:**
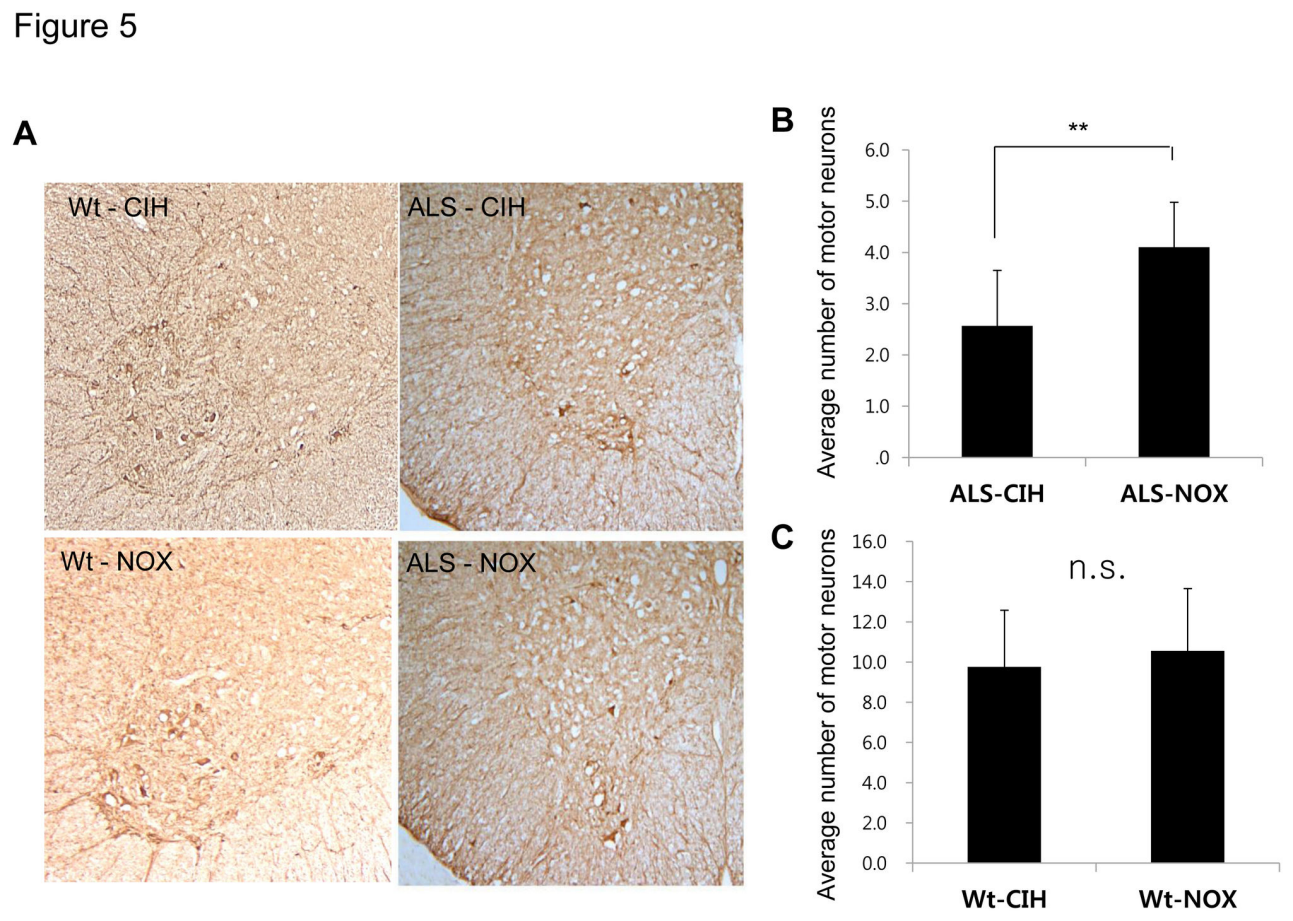
Immunohistochemistry with anti-choline acetyltransferase (ChAT) to assess the numbers of ventral horn motor neurons. Motor neurons in the ventral spinal cord were labeled with ChAT staining (A), and the numbers of motor neurons were counted in ALS mice. Significantly fewer motor neurons were counted in the ALS-CIH mice than in the ALS-NOX mice (B). The numbers of motor neurons did not differ significantly between the Wt-CIH mice and the Wt-NOX mice (C). **, *p* < 0.01, n.s. = no statistical significance.

The numbers of motor neurons in the ventral horn of the spinal cord were significantly lower in the ALS-CIH mice than in the ALS-NOX mice (2.57 ± 1.08 vs. 4.10 ± 0.88 neurons per ventral horn; *p* < 0.001). The numbers of motor neurons did not differ significantly between the Wt-CIH mice and Wt-NOX mice (9.77 ± 2.81 vs. 10.56 ± 3.10 neurons per ventral horn; *p* = 0.453). 

### CIH can provoke oxidative stress and activate the inflammatory pathway in ALS mice (Figures 6, 7, and S1)

**Figure 6:**
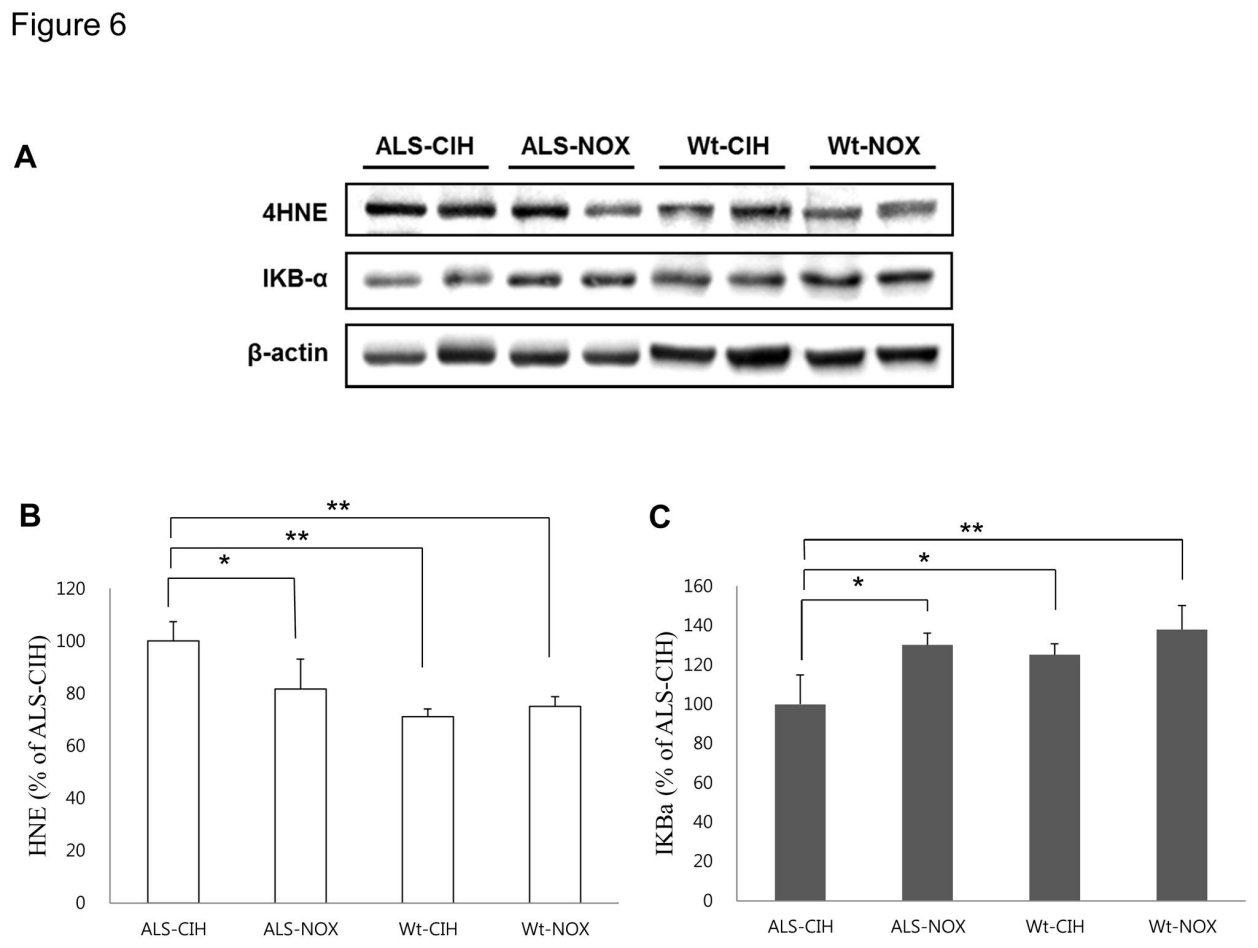
Western blot for markers of oxidative stress (5-HNE) and activation of the NF-κB pathway (IκBα). Generation of reactive oxygen species and/or activation of inflammatory pathways might be involved in the increased loss of motor neurons in ALS mice due to CIH. Although the mild CIH (minimum F_i_O_2_ of 7.8%) used in this study did not induce oxidative stress or activation of the NF-κB pathway in the Wt mice, significant oxidative stress (A and B) and activation of the NF-κB (A and C) pathway were demonstrated in ALS mice. * *p* < 0.05, **, *p* < 0.01.

**Figure 7:**
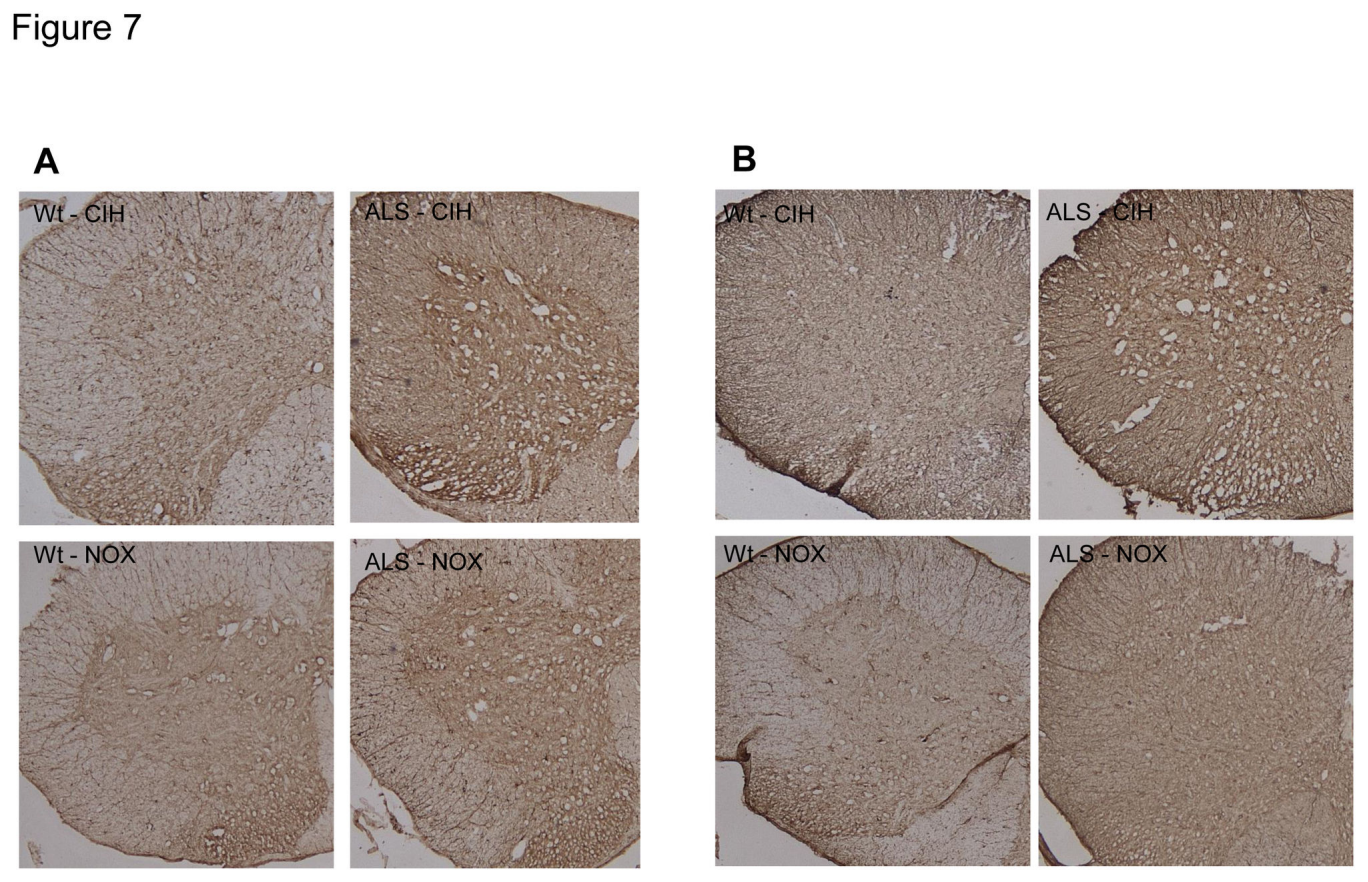
Immunohistochemistry (IHC) for astrocytosis and activation of microglia. Astrocytosis (A) and activation of microglia (B) were assessed using IHC assays for glial fibrillary acidic protein (GFAP) and ionized calcium-binding adapter molecule 1 (IBA1), respectively. Compared to the ALS-NOX and Wt mice, the ALS-CIH mice showed higher expression of both GFAP (A) and IBA1 (B).

Compared to the ALS-NOX mice, the ALS-CIH mice had significantly higher expression of 4-HNE (*p* = 0.004). In addition, although the expression of 4-HNE was only slightly higher in the ALS-NOX mice compared to the Wt-NOX mice (*p* > 0.05), the ALS-CIH mice had a significantly higher level of 4-HNE expression compared to the Wt-CIH mice (*p* < 0.001). Compared to the Wt-NOX mice, the ALS-CIH mice also had significantly higher levels of 4-HNE (*p* < 0.001) (Figure 6 A and B). Compared to the ALS-NOX mice, the ALS-CIH mice had significantly lower levels of IκBα, representing higher activation of the NF-κB pathway (*p* = 0.004). Although the ALS-NOX and Wt-NOX mice did not differ significantly in terms of IκBα levels, the ALX-CIH mice had significantly lower levels of IκBα compared to the Wt-CIH mice (*p* = 0.007). Compared to the Wt-NOX mice, the ALS-CIH mice also had lower levels of IκBα (*p* < 0.001) (Figure 6 A and C). Astroglyosis and activation of microglia were more prominent in the ALS-CIH mice than in the ALS-NOX and Wt mice, which was demonstrated by immunohistochemical analysis for GFAP (Figure 7 A) and IBA1 (Figure 7 B).

## Discussion

This study shows that CIH can impair motor learning and spatial memory function, accelerate the degeneration of motor neurons in the ventral horn of the spinal cord, worsen the progressive neuromuscular weakness, increase the synthesis of reactive oxygen species (ROS), and activate the NF-κB pathway in ALS mice.

This is the first study to show that hypoxia can affect motor neuronal loss, neuromuscular weakness, and probably cognitive dysfunction in an in vivo model of ALS. Until recently, numerous studies on ALS have proposed variable relationships between hypoxia and disease progression, including (1) supportive treatment with non-invasive ventilation prolonging the survival of patients with ALS [6]; (2) occupational conditions that can lead to intermittent hypoxia as a possible risk factor for ALS [23]; (3) selective impairment of the molecular response to hypoxia in ALS mice [12]; and (4) hypoxia, combined with hypoglycemia, leading to aggravated motor neuronal degeneration in an in vitro model of ALS [10]. However, no study has previously showed that hypoxia (either CSH or CIH) can actually aggravate motor neuronal loss or cognitive dysfunction in vivo models of ALS. Furthermore, a previous study using CSH failed to demonstrate an effect of hypoxia on disease progression in an in vivo model of ALS [10].

Discrepancies between the results of previous studies on the effects of hypoxia by using in vitro models, in vivo models, and in patients with ALS may primarily stem from the different types of hypoxia used in each study (e.g., use of only a CSH model or ischemic hypoxia [hypoxia combined with hypoglycemia] model, rather than the CIH model) [10,12]. The particular type of hypoxia used in each study is important because diverse hypoxic models have been proposed to result in different molecular responses [14,24]. For example, following CSH, the major molecular mechanism proposed has been the stabilization of hypoxia inducible factor-1 alpha (HIF-1α) and subsequent increase in the transcription of erythropoietin and/or vascular endothelial growth factor. In contrast, CIH primarily activates the NF-κB pathway and up-regulates the synthesis of ROS [14]. 

The results of this study also suggest that ALS mice might be more vulnerable than Wt mice to CIH based on the following evidence. This study used a model of mild CIH, in that the CIH conditions in this study reached a minimum F_i_O_2_ of 7.8%, which is less severe than conditions used in previous studies with a model of obstructive sleep apnea (minimum F_i_O_2_ of 5%) [25]. Under this mild CIH, ALS mice, but not control mice, showed significant cognitive dysfunction, synthesis of ROS, and activation of inflammatory pathways.

**Figure 8:**
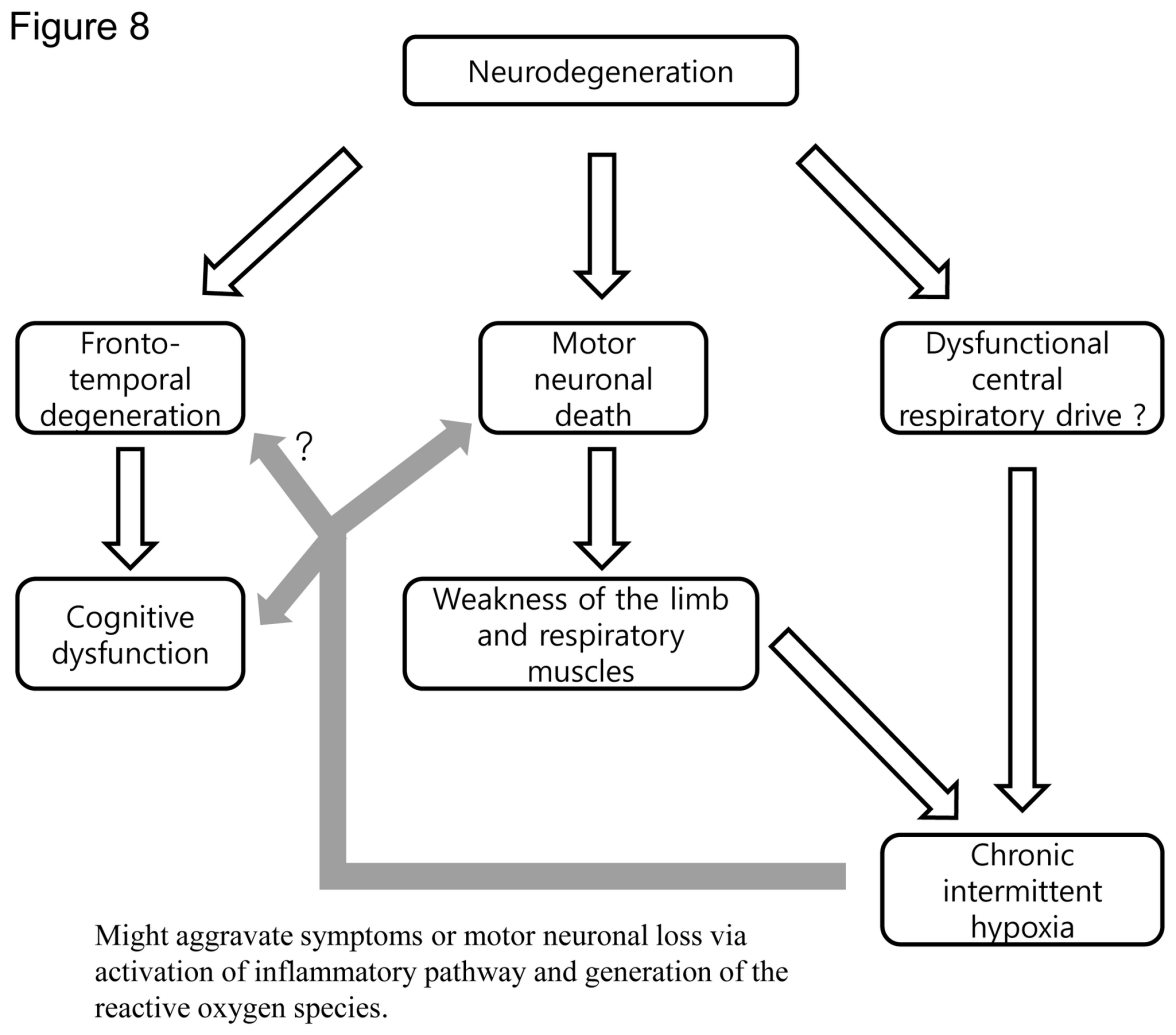
Diagram showing the possible effects of CIH in ALS. Two main features of ALS, cognitive dysfunction and motor weakness, are due to the degeneration of the central nervous system. The symptom of motor weakness, most likely accompanied by dysfunctional central respiratory drive, can cause CIH in patients with ALS. CIH, in turn, might aggravate motor neuronal loss and cognitive dysfunction in ALS via the generation of reactive oxygen species and/or activation of inflammatory pathways.

Conditions of CIH can be relatively common in patients with ALS. Previous studies have shown that 17–64% of patients with ALS had periodic (intermittent) nocturnal desaturations [8,26]. Another study also showed that patients with ALS had 10 times more episodes of apnea/hypopnea during sleep, implying that these patients had frequent episodes of intermittent nocturnal hypoxia [27]. Recent study also showed that the nocturnal hypoxia in patients with ALS can occur as clusters of desaturations [28]. The exact cause of this intermittent hypoxia in ALS remains unclear, but a previous study using polysomnography suggested that de-regulated central respiratory drive or respiratory muscle fatigue, rather than obstructive sleep apnea, were likely responsible for the intermittent nocturnal hypoxia [27]. We speculate that this intermittent hypoxia in patients with ALS might, in turn, aggravate their symptoms or signs via the generation of ROS or the activation of inflammatory pathways (Figure 8). 

The results of this study must also be viewed in light of the following limitations: (1) The number of mice in each group was relatively small (n = 8). (2) In this study, the CIH or NOX conditions were initiated in mice at the age of 14 weeks. This method was used because the symptoms of ALS in this mouse model begin at a mean age of 74 days after birth. Similarly, patients with ALS are only exposed to CIH after the initiation of symptoms of ALS [29,30]. Therefore, it is likely that this experimental paradigm closely resembles the actual clinical situation in patients with ALS. However, despite parallels between the current experimental design and the clinical reality of ALS, the outcome measures in this experiment were obtained at an advanced disease stage, which may have blunted differences between study groups and controls. The blunted differences due to an advanced disease state, combined with the relatively small number of our mice in each group, may also be responsible for the absence of statistical significance between groups in the rotarod test on Days 12 and 15, despite the significant difference in the number of the motor neurons and neuromuscular strength between ALS-CIH and ALS-NOX (3). Though we have evaluated the molecular response in the spinal cord of mice, we did not assess the pathological changes in the fronto-temporal lobes [5] of these mice. Further study with the detailed pathologic evaluation is needed to reveal the exact effect of the CIH in the cognitive dysfunction of ALS (4). We did not test wire-hanging time in our Wt mice, which if done, might have produced another interesting result (5). Although we showed that CIH down-regulated IκBa, an upstream target of NF-κB activation, we did not directly show the activation of the NF-κB gene.

Nevertheless, to our knowledge, this is the first study to demonstrate that CIH can aggravate motor neuronal loss and probably cognitive dysfunction in an in vivo model of ALS. Our study will provide insight into the association of hypoxia with the aggravation of the motor neuronal death and cognitive dysfunction in ALS, clues for determining the pathomechanism of ALS, and a rationale for the development of an early non-invasive ventilation treatment in ALS. Further studies will be needed to uncover the exact mechanisms of these findings, to understand the implications of these findings for unraveling the disease mechanism, and to identify applications for clinical practice.

## Supporting Information

Figure S1 **Western blot for markers of oxidative stress (5-HNE) and activation of the NF-κB pathway (IκBα).** Results were shown in four ALS-CIH mice, 3 ALS-NOX mice, 4 Wt-CIH mice, and 4 Wt-NOX mice.
(TIF)
