# Supplementary figures and images for: Intermittent Hypoxia Can Aggravate Motor Neuronal Loss and Cognitive Dysfunction in ALS Mice
Source: PLoS One. 2013 Nov 26;8(11):e81808. doi: 10.1371/journal.pone.0081808 (PMC3841127; doi:10.1371/journal.pone.0081808)

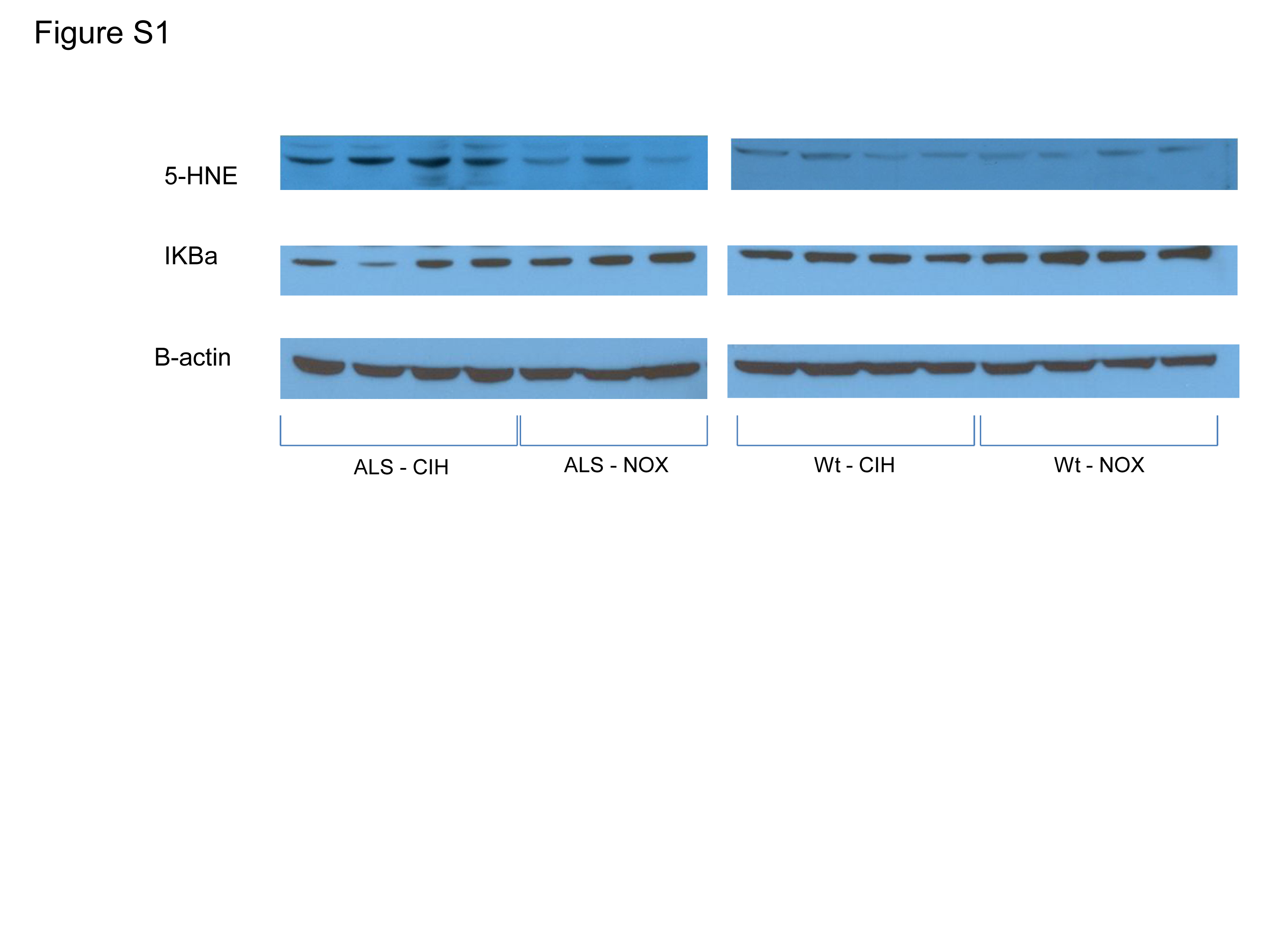

Supplement: Figure S1 — Western blot for markers of oxidative stress (5-HNE) and activation of the NF-κB pathway (IκBα). Results were shown in four ALS-CIH mice, 3 ALS-NOX mice, 4 Wt-CIH mice, and 4 Wt-NOX mice. (TIF) [file pone.0081808.s001.tif]
